# The culmination of multidrug-resistant efflux pumps vs. meager antibiotic arsenal era: Urgent need for an improved new generation of EPIs

**DOI:** 10.3389/fmicb.2023.1149418

**Published:** 2023-04-17

**Authors:** Shiela Chetri

**Affiliations:** Department of Microbiology, Thassim Beevi Abdul Kader College for Women, Kilakarai, Tamil Nadu, India

**Keywords:** efflux pump, MDR, antibiotic resistance, resistance nodulation division superfamily, efflux pump inhibitors (EPIs), *Escherichia coli*

## Abstract

Efflux pumps function as an advanced defense system against antimicrobials by reducing the concentration of drugs inside the bacteria and extruding the substances outside. Various extraneous substances, including antimicrobials, toxic heavy metals, dyes, and detergents, have been removed by this protective barrier composed of diverse transporter proteins found in between the cell membrane and the periplasm within the bacterial cell. In this review, multiple efflux pump families have been analytically and widely outlined, and their potential applications have been discussed in detail. Additionally, this review also discusses a variety of biological functions of efflux pumps, including their role in the formation of biofilms, quorum sensing, their survivability, and the virulence in bacteria, and the genes/proteins associated with efflux pumps have also been explored for their potential relevance to antimicrobial resistance and antibiotic residue detection. A final discussion centers around efflux pump inhibitors, particularly those derived from plants.

## Introduction

Bacteria are already well known for their ability to persist in extreme environments *via* their evolving numerous defense mechanisms against various substances that are lethal for their survival. Currently, antimicrobial resistance, the most complicated health concern across the world, which is primarily associated with an extended hospital stay, expensive treatment, and high mortality and morbidity rates, needs undeniable attention and multiple operative approaches involving the “One Health” concept. As the concept specifies, the interrelatedness among food, animals, and human health with the environment and the intentional collaboration between different sectors' efforts to confront the standing challenge (Taneja and Sharma, 2019), but regrettably, of antimicrobial resistance (AMR) development due to environmental influences have received less care as compared to human and animal health in spite of the significant deviations in the geographical dissemination of AMR associated with environment (O'Neill, 2015). The ultimate result of the increasing incidences of AMR is the development of multidrug-resistant phenotypes, and the multidrug-resistant (MDR) bacteria is a serious problem worldwide (Cooper and Shlaes, 2011; Blair et al., 2014; O'Neill, 2015; Venter, 2019). Moreover, unexpectedly, this problem occurs at the same time when several pharmaceutical companies stopped investing in research to find new drugs to treat MDR due to a lack of high financial returns. In the bacterial cell membrane, the presence of transporters helps in the extrusion of noxious compounds, ultimately decreasing the concentration of external and unwanted or irrelevant substances and enhancing growth. However, the role of some transporters in the biodegradation of various deadly substances present in our environment is also well known (Ganas et al., 2007). As per the previous reports available, there are various resistance mechanisms contributing toward the development of resistant phenotypes—both extrinsic and intrinsic (Ganas et al., 2007). However, the role of intrinsic mechanisms such as the overexpression of multidrug-resistant efflux pump systems in evolving MDR phenotypes has gained partial attention (Nikaido and Pagès, 2012). The role of these efflux pumps is not only limited to functions as an efficient transporter but also limited to combat the stress from external environmental for bacterial survivability. However, it should be noted that some efflux pumps are substrate specific while others have a broad range of substrate specificity, which means that they can pump out multiple substrates including different classes of antibiotics, thus prompting multidrug resistance in bacteria (Nikaido and Pagès, 2012). Therefore, the efflux pump systems which form an important antibiotic resistance mechanism are widely considered due to their unique ability to extrude a variety of noxious compounds including dyes, detergents, different classes of antibiotics, and disinfectants (Nikaido and Pagès, 2012; Venter et al., 2015) outside the cell and thus are intensely associated with MDR expansion. Moreover, these specific features of the efflux pump systems have made them more prominent as multidrug efflux pumps (Webber and Piddock, 2003; Piddock, 2006a).

For the survivability of bacteria, these transporters located in the bacterial cell membrane play a crucial role in facilitating the reduction of external and irrelevant substance concentrations and promoting bacterial growth. However, evidence indicating the role of these transporters in the biodegradation of deadly compounds that are present in the environment has previously been reported (Ganas et al., 2007), suggesting the role of these efflux pump systems not only as a transporter but also in defending bacterial growth and survivability under various environmental stresses. Substrate specificity of these efflux pumps against various substrates, such as different antimicrobial classes, induces the pathogens to become multidrug resistant (Hernando-Amado et al., 2016). However, in some cases it has also been observed that the ejection of the substrates or organic solvents results into overexpression of transporters, which further, triggers the co-selection of the altered features of antimicrobial resistance in bacteria (Blair and Piddock, 2016). As a consequence of overexpression of the efflux pump systems, biofilm formation and quorum sensing (QS) will also be impacted by bacterial pathogenicity (Alcalde-Rico et al., 2016; Kong et al., 2017). In addition to antimicrobials, efflux pumps also have the potential to export virulence determinants, such as adhesins, toxins, and other crucial proteins, which are very critical for colonization within host cells (Piddock, 2006b). Several studies have already been reported on unfolding the development of antibiotic resistance associated with efflux pump systems along with numerous novel efflux pump transporters and simultaneous proteins. Additionally, in recent years, several other functions of an efflux pump system besides antibiotic resistance, such as virulence and bacterial self-protection against various environmental pollutants, were investigated. However, the particular mechanisms of regulation of these efflux pump systems and the active domains of the transporters are still not clearly understood. There are several inducing factors located on the inner and outer membranes of bacteria that stimulate the activity of efflux pump systems and promote their structural alterations within the fluid membrane environment. A brief overview of the role of efflux pumps in Gram-positive and Gram-negative bacteria is presented in this review article. Furthermore, the applications of efflux pump regulators to identify antimicrobial resistance and antibiotic residues, as well as the discovery of new efflux pump inhibitors derived from plants, have been discussed in this study.

## Efflux pump families

**Figure 1:**
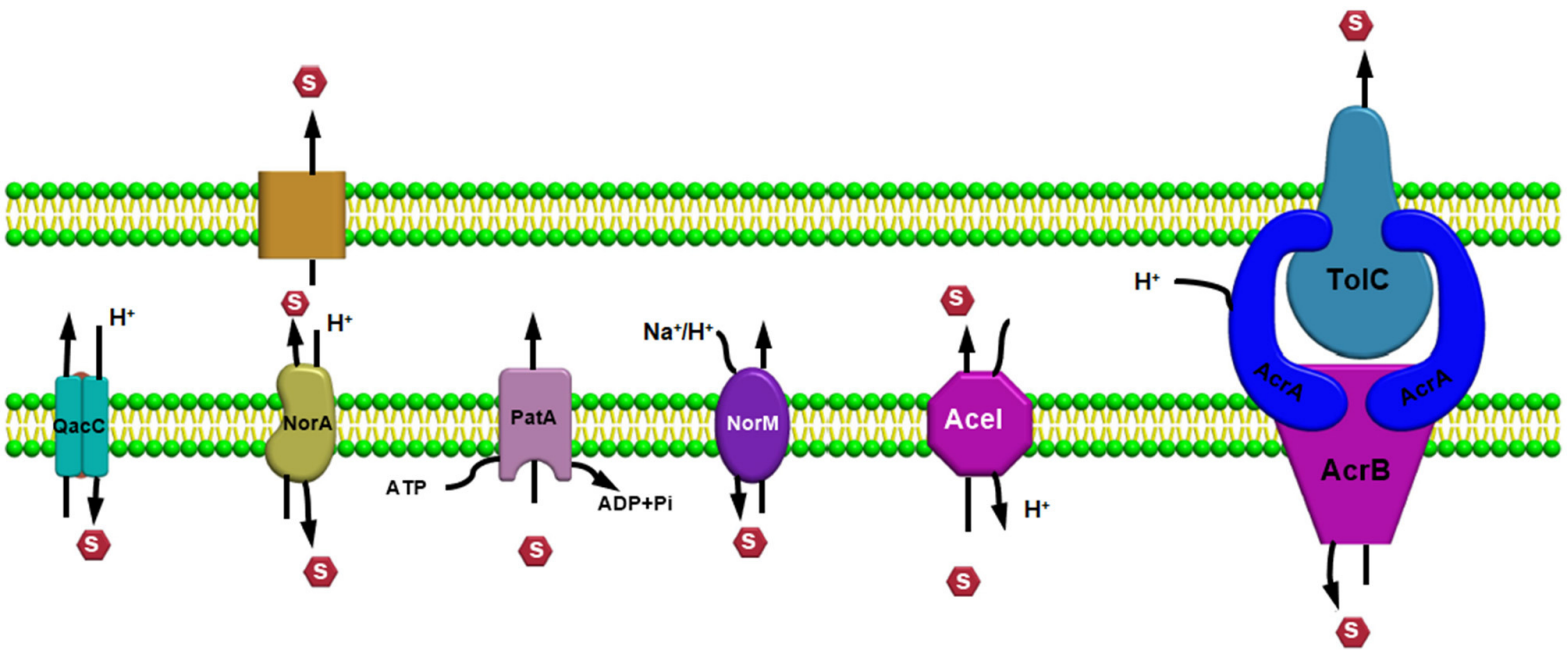
Efflux pump major super-families based on their energy source utilized to export the substrates.

Efflux pump systems are predominantly classified into six major superfamilies based on their energy source utilized to export the substrates: ATP-binding cassette (ABC) superfamily, multidrug and toxic compound extrusion (MATE) superfamily, major facilitator superfamily (MFS), resistance nodulation and cell division (RND) superfamily, small multidrug resistance (SMR) superfamily, and proteobacterial antimicrobial compound efflux (PACE) (Hassan et al., 2018) (Figure 1). One of the chief differentiating features of these efflux pumps is the source of energy they utilize; for example, the ABC superfamily draws energy *via* ATP hydrolysis (Verchère et al., 2012); however, other EP families such as RND, MFS, SMR, and MATE utilize either proton motive force (PMF) derived from H+ or electrochemical gradient of Na+ for exporting their substrates and for extruding several compounds (Kim and Hummer, 2012). Additionally, there are some other features as well for differentiating these efflux pumps based upon their composition or structure of the efflux pump transporters; for example, in an RND efflux pump system, three components, namely, an outer membrane protein (OMP) channel, an inner membrane transporter protein, and two modules that were connected *via* a membrane fusion protein (MFP) combine to form a composite known as a tripartite complex, which ultimately works together for the extrusion of noxious agents (Daury et al., 2016; Neuberger et al., 2018). However, research on novel multidrug-resistant (MDR) efflux pump systems in bacteria is still underway.

## ATP-binding cassette superfamily

The MacAB-TolC efflux pump system is a tripartite complex belonging to the ABC and is the most comprehensively studied EP system among gram negative bacteria which is actively associated with the extrusion of macrolides and polypeptides virulence factors driven *via* ATPase MacB and further participates in enterotoxin heat stable enterotoxins (TII) secretion in *Escherichia coli* (Lu and Zgurskaya, 2013; Fitzpatrick et al., 2017; Jo et al., 2017). Additionally, lipopolysaccharides (LPS) and analogous glycolipids can be considered as organic substrates of the MacAB-TolC pump systems (Lu and Zgurskaya, 2013). As the MacAB-TolC (pump system) is a tripartite complex with three components, including MacB, which is an inner membrane protein that functions as a homodimer complex and consists of two domains, namely, the (i) N-terminal, a nucleotide-binding domain that enables power generation *via* ATP hydrolysis and the (ii) C-terminal cytoplasmic tail (Lu and Zgurskaya, 2013). Other important components of the EP system are MacA, a periplasmic adaptor protein (PAP) that gets stimulated when ATPase binds specifically with the lipopolysaccharide core, and an outer membrane channel protein TolC that functions as an exit duct for substrate transport (Lu and Zgurskaya, 2013; Fitzpatrick et al., 2017). We observed from previous literature that, in *Serratia marcescens*, the absence of the MacAB (efflux pump system) resulted in increased susceptibility to polymixins and aminoglycosides, with a significant decrease in swimming motility and biofilm formation ability, and even caused impairment in defense capability to combat superoxide stress (Shirshikova et al., 2021). In addition, in their research study, Shi et al. (2019) observed that, in *Agrobacterium tumefaciens* 5A {a strain that is arsenite [As (III)] resistant}, MacAB efflux pump confers resistance against a variety of macrolide and penicillin-like antibiotics along with arsenite [As (III)].

However, Gram-positive bacteria harboring the ABC efflux pump systems, containing single component or transmembrane protein, have also been observed: for example, EfrAB transporter in *Enterococcus faecalis*, Msr protein in *Streptococcus*, LmrA in *Lactococcus lactis* (Hellmich et al., 2015), and PatA/B in *Streptococcus pneumoniae* (Garvey et al., 2011a; Iannelli et al., 2014). EfrAB, an MDR efflux pump system, is a heterodimeric protein conferring resistance toward different antibiotics such as gentamicin, chloramphenicol, and streptomycin (Lerma et al., 2014). In addition, the expression of the EfrAB pump was found to be highly induced when exposed to these antibiotics in sub-inhibitory concentration, and a decline in the expression was observed in the presence of a sub-inhibitory concentration of EDTA (3 mM) (Lerma et al., 2014). The LmrA transporter protein in *L. lactis* is a homodimer protein having one nucleotide-binding domain and six α-helix domains, which initially identifies and then proceeds toward the extrusion of macrolides and lincosamides (Hellmich et al., 2012). In *Streptococcus*, the Msr protein, comprising only two nucleotide-binding domains [without any transmembrane domain (TMD)], was found to be responsible for conferring resistance against macrolides and to collaborate with the Mef transport family (Zhang et al., 2016; Tatsuno et al., 2018). Further, in the case of the PatA/B efflux pump system, nearly the entire hydrophilic fluoroquinolone family members, such as ciprofloxacin, function as a substrate for this pump (Baylay and Piddock, 2015).

## Multidrug and toxic compound extrusion superfamily

Members of the multidrug and toxic compound extrusion (MATE) family are broadly disseminated among the three domains, namely, archaea, bacteria, and eukarya (Kuroda and Tsuchiya, 2009). This class of transporters belongs to the multidrug/oligosaccharide-lipid/polysaccharide (MOP) family, which is further categorized into two subfamilies, namely, (i) NorM and DinF found in prokaryotes and (ii) the eMATE subfamily in eukaryotes based on their sequence similarity (Brown et al., 1999; Omote et al., 2006). MATE transporter proteins in bacteria are mainly involved in the extrusion process of amphiphilic cationic drugs, such as norfloxacin, outside the cell. However, in plants, these transporters are involved in physiological roles like developing resistance toward herbicides, leaf senescence, auxin synthesis, tolerance against aluminum, homeostasis, and sequestration of organic compounds (which are plant-based) inside the vacuoles (Remy and Duque, 2014). Further, the MATE transporters were found to be located in both the proximal (convoluted and straight) tubules in the kidney in the case of mammals and in the canalicular domain inside hepatocytes, where the organic compounds are transported across this membrane domain during the final round of drug elimination process (Omote et al., 2006; Terada and Inui, 2008).

All life forms on earth need a balanced extrusion of exogenous noxious substances outside to sustain homeostasis. Therefore, the extrusion of lethal compounds such as xenobiotics requires the efficient involvement of the transporters. First, MATE family transporters were known for their ability to develop antibiotic resistance in *Vibrio parahaemolyticus* (Morita et al., 1998) but they later became well-known in other domains as well. Moreover, MATE is abundant throughout the human body, but its expression is highly concentrated in the bile canaliculi of the liver and within the brush border membrane of the kidney (Otsuka et al., 2005). Metformin, cimetidine, procainamide, and acyclovir (Masuda et al., 2006; Tanihara et al., 2007) are the cationic drugs that are recognized by the MATE transporter, and therefore, they act as influential factors in controlling plasma concentrations. However, previous literature suggests the significant role of MATE in inducing drug-associated nephrotoxicity during the last phase of pharmacodynamics of cationic drugs. An example is cisplatin, a clinically vital anticancer agent which has not been recognized by the MATE efflux transporter and has ultimately resulted in nephrotoxicity due to renal accumulation (Yokoo et al., 2007). Moreover, cimetidine, which is a histamine (H2) receptor antagonist that is a well-known MATE inhibitor functioning as a gastric acid inhibitor (Ito et al., 2012; Wagner et al., 2016), must be carefully handled and administered. Therefore, a deep understanding of the MATE transporter's working mechanisms is necessary for refining the efficiencies of cationic drugs. The MATE transporters are energetically involved in several physiological functions, which makes them attractive pharmaceutical targets. The MATE transporter protein structure is composed of 12-transmembrane helices arranged in two six-transmembrane (TM) domains, namely, the N- and C-lobes.

However, the positioning of these helices is arranged in an altered manner so as to make it different from the transporters belonging to the major facilitator superfamily (MFS) (He et al., 2010; Van Veen, 2010), supporting the previous assumptions that these two transporters should be considered as distinct superfamilies (Brown et al., 1999). Previous studies on the effective changes in the mechanisms, which are required either for the survivability of a particular microbe or for the development of a multidrug-resistant variant, have been observed in the case of a variety of transporter proteins. As a result of consecutive conformational variations, the transporter protein substitutes within two main conformations, one of which functions *via* exposing the substrate binding pocket (located centrally) to the inside and the other disclosures the pocket toward the outside of the cell. However, the structure and catalytic reactions mediated desired conformational changes are still not properly understood and one of the main reason behind this is the evidence indicating two crystal structures of the MATE transporter protein having an outward fronting conformation. Additionally, transporter proteins belonging to the MATE family were primarily well-known for mediating drugs or ions antiport *via* utilizing Na+ or H+ energy sources. The difference in the utilization of different energy sources such as H+ or Na+ gradient in different family members within the MATE superfamily has been suggested according to previous x-ray crystallographic studies concerning the NorM subfamily depending upon Na+ coupling in the C-lobe (He et al., 2010; Lu et al., 2013a), and a quite similar pattern was noticed in case of the eMATE subfamily (Miyauchi et al., 2017; Tanaka et al., 2017) but the DinF subfamily members was found to utilize H+ in the N-lobe (Lu et al., 2013b; Tanaka et al., 2013; Radchenko et al., 2015; Mousa et al., 2017; Kusakizako et al., 2019).

## Major facilitated superfamily

**Figure 2:**
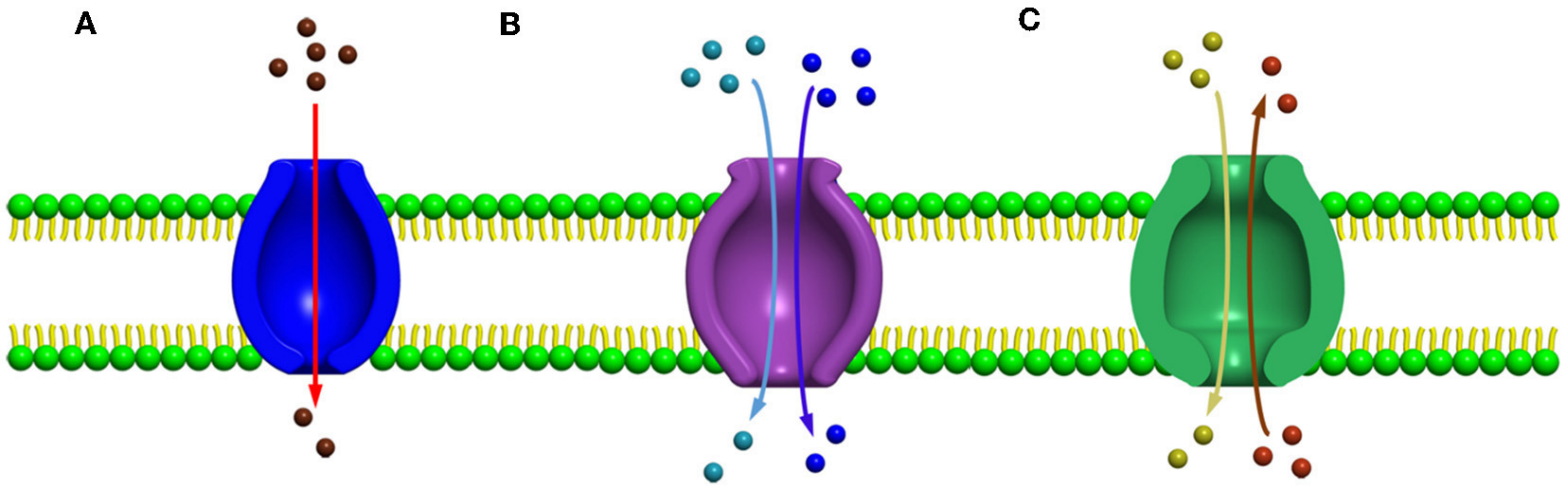
Major facilitator super family (MFS) transporters can be categorized into three main groups based upon their mode of transportation of their substrates **(A)** uniport, **(B)** symport, and **(C)** antiport.

Several secondary carrier families having similar topological features were identified before 1993, but no information was available to prove or to relate these families by a common origin. Among these families, a sugar porter's family including glucose facilitators found in mammals, two families of specific and multidrug efflux pump transporters, a metabolic uptake transporter family, and an oligosaccharide transporting family, including lactose permease present in *E. coli*, have been well studied. In addition, the knowledge and effort of bioinformatics helped us to understand the mechanisms and provided evidence to relate these families, and further, the name “major facilitator superfamily” (MFS) was coined (Marger and Saier, 1993). The major facilitator superfamily (MFS) transporters can be categorized into three main groups based on the mode of transportation of their substrates: (i) uniporters, which are responsible for the transference of a single substrate, (ii) symporters, which transport a substrate along with the simultaneous transportation of a coupling ion (e.g., H+), and (iii) antiporters, which are responsible for the simultaneous transportation of two substrates in opposite directions in such a way that the transportation of one substrate is dependent on prior discharge of the co-substrate (Forrest et al., 2011) (Figures 2A–C). In addition, it has been observed that the uniporters are able to transport their substrates without utilizing any energy source below their concentration gradient, while in the case of antiporters and symporters, the stored energy of the coupling ion concentration gradient is utilized for substrate transportation against their concentration gradient. However, irrespective of the differences in the mode of transportation of the substrates, all the members belonging to the major facilitator superfamily have the identical core structure comprising 12 TM helices, which are organized on two domains, namely, the N-domain and the C-domain (having similar structure) (Hirai et al., 2002; Yan, 2013), and are further partitioned into two inverted repeats of three helices.

One of the most well-studied MFS transporters in bacteria is the lactose permease (LacY) present in *E. coli*, which has worked as an appropriate protein model, for understanding the diverse features of the mechanisms involved in transportation associated with MFS transporters (Guan and Kaback, 2006; Smirnova et al., 2011; Kaback, 2015). However, in humans, the most studied MFS efflux pump (EP) system is the glucose transporter which plays a very crucial role in maintaining glucose homeostasis in the human body, which is also referred to as the SLC2 protein. Moreover, the deterioration or faulty regulation of these proteins may lead to important diseases such as diabetes (Type II) (Augustin, 2010; Cura and Carruthers, 2012). Further, the roles of the Msr transporter belonging to the ABC superfamily and the Mef system of the MFS family were found to increase the macrolides extrusion co-actively, which further enhanced the resistance toward 14–15-member ring macrolides (Nunez-Samudio and Chesneau, 2013). Besides their active participation in the extrusion process, these MFS transporters are also linked with other biological pathways, such as the role of MdrT and MdrM in promoting the immune response in hosts by activating the production of interferon-β (IFN-B, which is a type I interferon response) and in maintaining the stability of the cell wall (Pasqua et al., 2021). In the case of *Staphylococcus aureus*, the presence of a constitutively expressing MFS efflux pump system Tet38 has been reported to influence the progression of host cell invasion comprising internalization, adhesion, and relocation within epithelial cells and the consequent step involved in epithelial cell infection due to *Staphylococcus aureus*, which includes the viability of bacteria and trafficking within phagolysosomes (Pasqua et al., 2021).

However, in *Acinetobacter baumannii*, loss of the MFS transporter protein AbaQ resulted in a significant decrease in bacterial virulence and motility capability (Pasqua et al., 2021), and furthermore, a substantial reduction in bacterial motility and virulence was detected when the efflux pumps encoding genes (such as RND, MATE, SMR, and ABC) are inactivated (Pérez-Varela et al., 2019). The working mechanisms of MFS transporters still require more explanation. However, an updated form of the classical rocker–switch model for understanding the changes emerging in the conformation of the MFS transporter associated with a form of substrate transportation known as the “clamp-and-switch” model has been previously proposed (Quistgaard et al., 2016), which also discussed the function of gating residues, binding of substrates, and the conformational change observed in the updated model (Quistgaard et al., 2016). In the past decades, many molecular biology, biochemical, and biophysical experiments have been conducted to understand the function of MFS transporters due to their physiological and pharmacological significance.

## Resistance nodulation division superfamily

**Figure 3:**
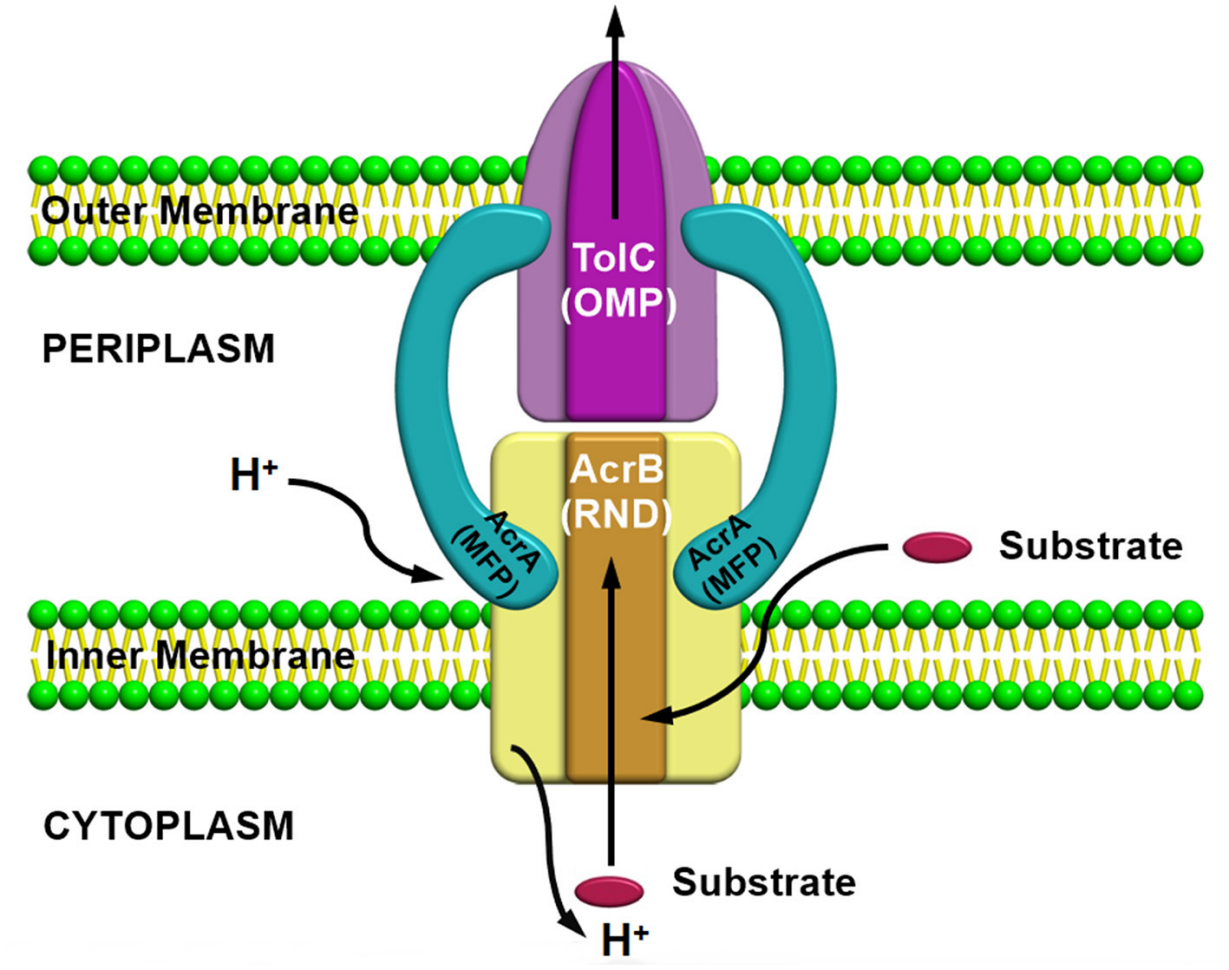
AcrAB-TolC a tripartite efflux pump system containing three components together to form a complex.

Resistance nodulation division (RND) superfamily members are the most well-known efflux pump systems because of their astonishing capability of extruding a wide range of substrates, which includes various classes of antibiotics, such as tetracyclines, chloramphenicol, different β-lactam antibiotics, and besides antibiotics, several other compounds such as dyes, detergents, metals, bacterial metabolites are also utilized as a substrate by this pump (Munita and Arias, 2016). The only feature which is common among these compounds is the amphiphilic nature of the substrates utilized by this pump (Yamaguchi et al., 2015). RND family members are part of multiprotein complexes which are arranged within the bacteria in a way that connects the outer membrane, periplasmic space, and the inner membrane to form a tripartite complex; thus, the RND transporters can facilitate the transport of compounds into these compartments by allowing them to work in conjunction with other transmembrane transporters. RND transporters are capable of capturing substrates not only from the cytoplasm but also from the inner membrane's outer leaflets or from the periplasm (Du et al., 2015). Consequently, it has been observed that these protein complexes are larger than several other transmembrane proteins of bacteria, which usually range over only a single membrane. Resistance nodulation division (RND) family members is comprised of three components, including (i) a periplasmic adaptor protein (PAP) (ii) an outer membrane protein (OMP) channel, and (iii) an MFP, which connects IMP and OMP, and are referred as a tripartite system for having three components together to form a complex (Figure 3). The main function of this tripartite complex is to identify and bind the substrates found in the periplasmic space, outer leaflet of the inner membrane, or the cytoplasm and lastly extrude or expel outside the cell through the outer membrane channel. However, other transporter protein functions as a single unit within the inner membrane for transporting substrates *via* the membrane bilayer (Nikaido, 2011; Du et al., 2018). Besides the participation of efflux pumps in the development of antibiotic resistance, other mechanisms such as alteration in drug target or inactivation or modification of antibiotics also have a vital role in AMR (Walsh, 2003; Blair et al., 2015; Colque et al., 2020). However, it has been noticed that comparative to the single factor that contributes to the resistance development against a particular class or group of antibiotics, multidrug resistance (MDR) can progress due to a reduction in bacterial membrane permeability (Nikaido, 2009) and *via* overexpression of the MDR efflux pump system in both Gram-positive and Gram-negative bacteria (Nikaido, 2009; Allen et al., 2010; Li et al., 2015). Besides being intrinsically expressed or present, bacteria also acquire resistance by RND efflux pumps *via* plasmids, etc. (Levy and Marshall, 2004; Allen et al., 2010), and mutation is intensely associated with the significant rise in the expression level of both intrinsic and extrinsic efflux pump systems in the clinical strains (Blair et al., 2015). This resulted in an increase in the expression level, which is one of the chief reasons for MDR development (Blair et al., 2014). In the current scenario, resistance nodulation division (RND) superfamily members are considered among the core contributors to MDR progress in clinical isolates (Nikaido, 1996; Blair et al., 2014).

Besides, the ability of these RND transporters to work as a tripartite pump in the extrusion of a wide variety of substrates such as different classes of antibiotics, detergents (Zwama and Yamaguchi, 2018), dyes, and various other noxious compounds outside the cell (Du et al., 2014), the overexpression of the efflux pump genes as a result of an increase in the transcriptional level of the operonic genes due to mutations in their regulatory proteins and genes (Nishino et al., 2008, 2009) has already been well studied and established in pathogenic bacteria within clinical settings (Oethinger et al., 1998; Nishino et al., 2008, 2009; Blair et al., 2015; Yu et al., 2020; Salehi et al., 2021). Examples of some of the well-studied RND efflux pump systems observed in Gram-negative bacteria are AcrAB-TolC in *E. coli* and *Salmonella enterica*, MexAB-OprM in *Psedomonas aeruginosa*, and the AdeABC system in *A. baumannii* (Bhakdi et al., 1988; Nikaido, 1996; Xu et al., 2020; Lv et al., 2021) are intensely associated with the extrusion process of a wide range of lethal compounds. Other examples such as the CmeABC system in *Campylobacter jejuni*, SmeABC in *S. enterica*, and the MtrCDE efflux pump system in *Neisseria gonorrhoeae*, have also been reported previously (Gilson et al., 1990; Singh et al., 2006; Wehmeier et al., 2009; Guérin et al., 2014; Wright and Tate, 2015; Lv et al., 2021). An important role of the RND transporter proteins, such as MtrD, MexB, AcrB, and AdeB, within the system, is in the substrate binding step (where the substrate may be specific) and in the drug transportation process, which is crucial for resistance development clinically (Delmar et al., 2014). An example is the mutation in AcrB gene which resulted in ciprofloxacin treatment failure (B). According to the previous literature, the transcriptional level of the regulatory proteins belonging to the TetR family such as AcrR (Blanco et al., 2016; Chetri et al., 2019a), MtrR (Lin et al., 2017), CmeR (Zwama and Nishino, 2021), TtgR (Bay and Turner, 2009), MexR (Srinivasan and Rajamohan, 2013), NalC/NalD (Bay et al., 2008), SmeT (Lytvynenko et al., 2016), and RarA belonging to the AraC family (Chetri et al., 2018b) influences the expression of these efflux pumps. The amino acid (AA) residues within the efflux pump systems are very crucial and actively participate in the substrate binding course and the substitution, or any changes in the AA residues probably can modify the substrate affinity (Banigan et al., 2015). Further, alteration in prominent AA residues was demonstrated to associate with efflux pump-facilitated resistance toward various drugs (Padariya et al., 2018). AcrAB-TolC, MexAB-OprM, and CmeABC are among the most well-characterized multidrug resistant efflux pump systems belonging to the RND superfamily and are responsible for survivability and pathogenicity in bacteria. There are reports indicating the co-existence of different defense mechanisms including porin deficiencies and β-lactamase production with the overexpression of efflux pumps in *E. coli* (Chetri et al., 2019a,c), but rare evidence of insertions into penicillin-binding protein 3 (PBP3) of *E. coli* affecting their activity against monobactams and cephalosporins has already been reported (Alm et al., 2015). In carbapenem-resistant *E. coli* isolates, the overexpression of the AcrAB-TolC efflux pump simultaneously with the downregulation of OmpF due to regulation *via* the MarA gene was observed (Chetri et al., 2020). Further, for a comparative analysis study in carbapenem-resistant *E. coli* isolates, the β-lactamase gene (*bla*NDM-1, *bla*CTX-M-14, and *bla*CTX-M-3) were targeted and amplified (Shimizu et al., 2017; Chetri et al., 2019b). However, a study showing the capability of the AcrAB-TolC efflux pump system in influencing bacterial attachment, then penetration within the host, and further proliferation and persistence within animals has been reported (Piddock, 2006b) previously. Besides, the role of the AcrAB-TolC efflux pump in *E. coli*, AcrEF-TolC has also been reported to have a significant role in developing resistance toward different antibiotics (Chetri et al., 2018a).

Extensive usage of heavy metals (HME) in antimicrobials is not an infrequent event, for example, in disinfectants, and as a result, the RND efflux pump superfamily has developed the ability to resist these compounds for their survivability. Further, these RND superfamily is divided into two main categories: (i) hydrophobic and amphiphilic (HAE)-RND family, for example, AcrB, AcrD, AcrF, MdtC, MdtD, and YhiV, (ii) HME-RND family, for example, CusA which utilizes Cu(I) and Ag(I) as the most important substrate (Delmar et al., 2014), where CusA functions as a membrane fusion protein, CusC as an outer membrane channel protein to form a tripartite complex, the CusCBA efflux pump system (Long et al., 2012). However, the mechanical pathways required for the exportation of metal ions have previously been predicted (Long et al., 2012). Further, for survival, bacteria adapts in different ways such as efflux pump-associated biodegradation to get rid of the harmful effects of the lethal chemical compounds resulting from organic pollutants, for example, in *Pseudomonas Putida*, the occurrence of the TtgABC efflux pump system is responsible for conferring tolerance toward toluene (Blanco et al., 2016). Consequently, due to pressure exerted *via* overuse of antimicrobials, mutations in functional genes occurs, which may influence various changes in the AA residues located in the binding sites, which affect the substrate specificity of an efflux pump, and as a result, bacteria become less receptive toward antimicrobials, i.e., antimicrobial-resistant phenotype significantly altering the scenario in the environmental, clinical, and laboratory settings (Zwama and Nishino, 2021). The genetic changes occurring in an efflux pump denote an evolutionary adaptation within a microorganism against antimicrobials, and it is undoubtedly complexing the entire therapeutic option management system of pathogenic bacteria (Zwama and Nishino, 2021).

## Small multidrug resistance family

Small multidrug resistance (SMR) family members utilize proton gradient to export a wide range of antibiotics, hence contributing toward MDR development. On the basis of the sequence, the SMR family can be divided into two main physiological subtypes (Kermani et al., 2020): (i) guanidinium exporter (representative of “Gdx”) which is responsible for the allocation of bacterial metabolites such as guanidinium ion in exchange of two protons (Nelson et al., 2017; Kermani et al., 2018) and (ii) quaternary ammonium compound (Qac) representative subtype, which is responsible for the export of certain hydrophobic, cationic, and quaternary ammonium compounds (Nelson et al., 2017; Kermani et al., 2018). After the discovery of the first antiseptics containing quaternary ammonium around 100 years ago, Qac cluster proteins were found to be nearly associated with the propagation of multidrug resistance elements (Russell, 2002; Pal et al., 2015; Gillings, 2017; Zhu et al., 2017). Several bacteria harboring both subtypes of SMR transporter proteins were detected. Additionally, it was observed that the two subtypes do not interfere with each other's physiological role, for example, Qac protein will not participate in the transportation of guanidinium ion (Gdm+) and likewise, the guanidinium exporter will not involve in the export of any cationic or quaternary ammonium compounds but will require a substrate that is having guanidine moiety (Kermani et al., 2020). In *E. coli*, an example of the most well-studied Qac transporter protein is EmrE, and also an EmrE mutant, i.e., S64V that is having diminished conformational exchange dynamics based on extensive NMR measurements has been recently reported (Shcherbakov et al., 2021). SMR family members comprise short polypeptide chains of 100–150 amino acids and can stretch the cytoplasmic membrane as 4 TM α-helices (Bay et al., 2008). Proteins with short hydrophilic loops allow solubilization of a broad range of drugs, noxious lipophilic compounds that comprise DNA intercalating agents, lethal metabolites such as nicotine, and polyamine compounds such as spermidine (Bay et al., 2008; Bay and Turner, 2009). There are several reports indicating the active role of these family members in developing resistance toward different substrates—like in *A. baumannii*—AbeS transporter is found to be responsible for the transportation of ethidium, benzalkonium, and acriflavine, which simultaneously increases resistance toward amikacin (Lytvynenko et al., 2016; Lin et al., 2017). Similarly, in *Klebsiella pneumoniae* activation of the efflux pump system, KpnEF confers resistance against chlorhexidine, other antiseptics, benzalkonium chloride, etc. (Srinivasan and Rajamohan, 2013). In both *P. aeruginosa* and *E. coli*, the role of EmrE transporter protein in recognizing the substrates and mediating the elimination of polyaromatic compounds has already been reported (Banigan et al., 2015; Chetri et al., 2018b), but the Qac proteins are particularly associated in resistance development against different antibiotics and antiseptics (Jaglic and Cervinkova, 2012). Frequent incidences of QacA or QacB genes in *E. faecalis* and *S. aureus* have been observed, whereas in the case of *Enterobacteriaceae* and *Pseudomonas* spp., the dissemination of qacE gene was more prevalent (Jaglic and Cervinkova, 2012). SMR family members are plasmid-encoded and can often occur on integrons (the mobile genetic elements [MGEs]), which influences the menace of horizontal spread of resistance (Bay and Turner, 2009). Incidences of QAC exposure influencing the overexpression of the efflux pump supports the horizontal transfer of integrons or MGEs harboring fluoroquinolone resistance determinants within Class I intergron (QacED1) (Anandapadamanaban et al., 2016; Buffet-Bataillon et al., 2016). The transfer of both the antibiotic-resistant and disinfectant-resistant genes between diverse species regulates the bactericidal effect of these lethal compounds.

## Proteobacterial antimicrobial compound efflux family

The abundance of these efflux pump systems in proteobacteria has provided a purpose for the naming of the family as the proteobacterial antimicrobial compound efflux (PACE) family (Hassan et al., 2015a). One of the well-studied transporter proteins of the PACE family, *Acinetobacter* chlorhexidine efflux (AceI) found to be responsible for developing resistance toward different compounds and additionally, numerous homologs of AceI conferring resistance against various biocides and mediating transportation of fluorescent dyes, such as proflavine and acriflavine, has been observed which differentiates this family from other families of efflux pump (Hassan et al., 2015a). Presently, PACE family members responsible for the extrusion of around 10 substrates have been reported and the transporter proteins belonging to this family are included in the Database of Transporter Classification (Saier et al., 2016), which comes under proteobacterial chlorhexidine efflux (CHX) family (Elbourne et al., 2017). Transporter protein of the PACE superfamily has been reported for the export of biocides such as acriflavine and chlorhexidine; however, transporter AbgT having a role in the extrusion of sulphonamides was observed. Regulators play an important role in modulation the efflux pump systems for drug extrusion, and the factors influencing drug resistance such as under tetracycline exposure, TetR regulates TetB expressional level and in case of cationic antimicrobials pressure, QacR controls qacA expression (Grkovic et al., 2002). In bacterial membranes, the regulators control the systems in such a way that, when there is a requirement of efflux, and only the expression of the efflux pump will proceed; thus, avoiding lethal effect of overexpressed efflux pumps that are constitutively expressing within the bacteria and saving the cellular resources (Andersson and Levin, 1999). From a research viewpoint, the identification of the efflux pump (EP) system recognizing the substrates or novel factors associated with resistance or tolerance against drugs can be analyzed through observation of the changes occurring in their transcriptional level, and it is possible because of the constricted regulatory control of EP genes. The analysis of the transcriptional response of *A. baumannii* toward chlorhexidine (a membrane-active biocide) revealed the role of *Acinetobacter* chlorhexidine efflux protein (AceI) system in *A. baumannii* (Hassan et al., 2013, 2015b). Antibiotic-resistant strains of *A. baumannii* are becoming increasingly prevalent in hospitals because they have emerged as a major cause of Gram-negative infections.

## Function and regulation mechanism of efflux pump

Efflux pump genes are present in all species of bacteria, and the efflux pump encoding genes are located either on a chromosome or a plasmid, especially in clinical cases (Piddock, 2006b; Poole, 2007). The understanding of the regulation of these efflux pumps, finding their function, their participation in developing resistance toward specific or multiple substrates, and their response under different environmental conditions, all of which were very crucial for the implementation of an accurate action plan. For a variety of membrane transporters, alternating access mechanisms have shown evolutionary persistence over the past few decades (Jardetzky, 1966). During sequential conformational modifications, a substrate-binding pocket in the middle of the transporter is exposed to the inner cellular space, while the other exposes the pocket to the outer cellular space through multiple conformational changes (Raturi et al., 2021). However, the structure and catalysis of MATE transporters underlying conformational changes are still not clearly understood. Partly, this is due to the fact that, particularly, two previously reported crystal structures of MATE transporters were with an outward-facing orientation (Raturi et al., 2021). Therefore, the MATE transporters facilitating drug/ion coupled transportation *via* Na+ or H+ coupling (Lu et al., 2013a) have already been described. The functionally diverse ABC family members utilize ATP to export their substrates and some act as ion channel modulators (Li et al., 2017). The structure of the ABC family transporter consists of two domains—one with substrate binding pocket (TMDs) and another with nucleotide-binding domain (NBDs)—which binds and initiates hydrolysis of ATP to stimulate the transport cycle (Dawson and Locher, 2006; Jin et al., 2012; Choudhury et al., 2014; Kodan et al., 2014; Johnson and Chen, 2017; Verhalen et al., 2017). An “alternating access” mechanism involved in the transportation of the substrate has been observed based on the functional and structural data available, in which the transportation triggers the switch in the conformation between inward-open, occluded, and outward-open states required for substrate transportation, and additionally, these conformational alterations are associated with the dimerization and dissociation of the nucleotide-binding domain-mediated *via* binding and hydrolysis of ATP (Khare et al., 2009; Korkhov et al., 2012; Woo et al., 2012). Consequently, in *E. coli*, the presence of ABC transporter protein MsbA utilizing the ATPase activity to transfer lipopolysaccharide precursor lipid A from the inner-cytoplasmic membrane to the outer-periplasmic membrane has been observed (Woebking et al., 2005), and as a result of its catalytic action, this enzyme is referred to as a flippase. Further, the cryogenic electron microscopy (cryo-EM) studies of the structural aspects of MsbA protein helped to understand the functional states and the visualization of the transportation route (Mi et al., 2017). However, some ABC transporters have shown that the MacB exhibits only outward-directed transport mechanism for the substrate entering through the periplasmic side, and as a result, the state of the substrate binding pocket remains in the outward direction. The transporters have the ability to interfere with their substrates from the outside facing binding pocket followed by a conformational alteration coupled with ATP hydrolysis, which enables the reduction in the affinity toward the ligand inducing its shifting into the exterior part (Perez et al., 2015; Locher, 2016). Similar to the ABC transporter family, the mechanism of alternating access was also reported in MFS transporters as well where in the course of a transport cycle the conformational switch of the two domains within inward to outward open states has been reported (Jardetzky, 1966). According to the data obtained from lactose permease symporter (LacY), the conformational switch of the alternating access mechanism is activated *via* induced fit from the ligand binding and further, and the transport rate is governed by the electrochemical proton gradients (Kaback, 2015). The fundamental component of the model is the formation of a ternary complex when the proton and substrate bind in an ordered manner. However, various MFS transporters bind in different sequences as well, such as initiated with binding with proton, then substrate, or with a substrate first and then proton. Additionally, it has been observed that the transportation cycle of MFS protein generally involves the binding (as an initial step) followed by the release of substrate and proton, however, variations in the stoichiometric drug–proton interconversion within family members have been reported (Schaedler and Van Veen, 2010; Fluman et al., 2014). Small multidrug resistance (SMR) efflux pump system utilizes proton motive force as an energy source was previously reported *via* an experiment with *S. aureus* Smr (Sau-Smr) conducted first by Grinius and Goldberg in 1994 (Littlejohn et al., 1992; Grinius and Goldberg, 1994). Moreover, this experiment revealed that an electrochemical proton gradient was utilized as the energy source for tetraphenylphosphonium (TPP) efflux due to the reconstitution of Sau-Smr efflux within proteoliposomes. Further, under similar experimental conditions, similar results were obtained for Eco-EmrE, the SMR homolog (Yerushalmi et al., 1995, 1996). Therefore, SMR proteins are considered a proton-dependent MDR efflux pump system (Paulsen et al., 1996). Despite QACs, SMR proteins are able to transport neutral and negatively charged compounds (Jack et al., 2000; Nishino and Yamaguchi, 2001). However, previous studies revealed that the nature of the compound has a significant effect on the energy required for substrate transportation observed in Eco-EmrE studies, for example, the transportation of TPP requires movement of the charge for an active efflux besides the transportation of methyl viologen (MV) (a lipophilic divalent cation) (Yerushalmi et al., 1995; Rotem and Schuldiner, 2004). Further, as a result of several structural studies of the AcrB protein of *E. coli* by utilizing X-ray crystallography (Murakami et al., 2002; Nakashima et al., 2011; Eicher et al., 2012) and cryo-EM techniques (Du et al., 2014), the transporter protein was well-studied and became the most expansively characterized member of RND family (Zgurskaya, 2009; Zwama et al., 2019). Additionally, the interaction of AcrB with a wide range of substrates, which are chemically distinct, was analyzed to understand the poly-specificity feature of the transporter protein (Murakami et al., 2006; Nakashima et al., 2011; Eicher et al., 2012, 2014; Hung et al., 2013; Mousa et al., 2016; Sjuts et al., 2016; Zwama et al., 2018). Further, the movement of the substrates *via* the transporter is driven by the conformational changes taking place within the system, which is also revealed by the previous studies (Du et al., 2014; Blair et al., 2015). Moreover, the understanding of the function of these EP systems and the inhibition mediated *via* small molecules was simplified with the help of molecular simulation studies (Vargiu and Nikaido, 2012; Vargiu et al., 2018). The entire protein domains present were defined by the initial crystal structure developed for AcrB, which revealed that the region of transmembrane consists of 12 α-helices that anchor the inner membrane protein and supports proton movement and is accompanied by the transportation of the substrates and energy supply to drive translocation (Murakami et al., 2006; Seeger et al., 2006). Further, the entry of the substrates into the multiprotein complex is facilitated by the groove located between 8 and 9 transmembrane helices, which provide one out of two distinct access sites for the substrates, and the remaining protein residue projects into periplasm which is required for binding of the substrates and to link up with AcrA (periplasmic protein) as well as with the outer membrane channel protein TolC that is rooted in the outer membrane. The carrier domain of AcrB having the ability to span periplasm consist of four sub-domains, namely, PN1, PN2, PC1, and PC2, each of which comprises a common motif pattern, namely, two β-strand–α-helix–β-strand motif. The carrier sub-domains of all three protomers are arranged in such a way that a central pore along with a long channel extending through the membrane has been formed for the translocation of the substrate (Zwama and Yamaguchi, 2018).

Further, it has been reported that a second access site for substrate was found to be located in a cleft that formed in between PC1 and PC2 domains. The binding of AcrA and TolC is facilitated by the docking domains (DN and DC), which are located on the top of the porter domain. Additionally, tight binding of the top of AcrB with the TolC base is very essential for preventing the substrate leakage into periplasm aided by β-strands, which are placed antiparallel to generate a funnel-like construction. The crystal structure initially reported by Murakami et al. (2002) revealed the crystallized symmetrical homotrimeric structure without any bound ligand and explained the entire structural design of AcrB. According to three consequent studies published in 2006, it has been first reported that the establishment of an AcrB complex with various ligands that demonstrated the adoption of diverse conformations by each of the three protomers resulted in an asymmetrical homotrimeric structure assembly (Murakami et al., 2006; Seeger et al., 2006; Su et al., 2006), which has been indicated to be an active state. Therefore, under these circumstances, three different conformations have been well-defined as follows: (i) loose (L) that is mainly associated with the access for the substrates, (ii) tight (T) that is linked with the binding course, and (iii) open (O) state coupled with the extrusion conformation (Seeger et al., 2009; Nikaido, 2011; Eicher et al., 2012). As the substrates move over the protein, the monomer rotates between L, T, and O and returns back to L, and this “L conformer” which is accessible for substrate binding is also recognized as the resting state (Murakami et al., 2006; Seeger et al., 2006; Nakashima et al., 2011). First, the substrate enters into AcrB during the unliganded L state either from the periplasm or inner membrane envelope *via* one of the two defined entry sites, which are then released into a proximal binding pocket (PBP). Additionally, as a result of the movement of substrates *via* AcrB in the direction of the distal binding pocket (DBP), which is situated deep in the carrier domain, a conformational alteration toward the T state assisting the transfer of the substrate from the central pore of AcrB toward TolC entrance has been observed. Subsequently, the ejection of the substrate from AcrB into TolC occurs *via* the rotating functional mechanism, which involves the protein transitional step, i.e., from state T to state O (Murakami et al., 2006; Seeger et al., 2006). Under that situation, state O which indicates an open state, a channel which is generated for substrate extrusion from AcrB to TolC and during the transportation of substrate, and several compressions and hindrances occurred inside the porter domain which facilitated the transportation of substrate in a unidirectional way toward central funnel referred as “peristaltic pump mechanism” (Seeger et al., 2006, 2008; Pos, 2009). In AcrB, the substrate entry *via* the two primary sites, primarily, *via* the proximal binding pocket (PBP) and then toward the distal binding pocket (DBP), plays an important role in defining the poly-specificity feature of the transporter.

However, in the case of hydrophobic substrates which are separated in the inner membrane's outer leaflet can enter through the TMD, containing hydrophobic grooves and are well defined as TM8 and TM9 (Murakami et al., 2006; Pos, 2009). Thereafter, tunnel 1 is formed which then elongates from the periplasmic membrane plane at the end of the TM8/TM9 groove till it links the proximal binding pocket (PBP) 2. However, comparatively, the entry of compounds confined to periplasm occurs *via* another site placed within the porter domains PC1 and PC2, ~15 Å on top of the membrane plane, leads toward tunnel 2, which further elongates in the direction of the protein center (Eicher et al., 2012). Finally, after linking tunnels 1 and 2, it leads toward a distal binding pocket located deep within AcrB protein, where the presence of a glycine-rich switch loop that is proposed to contribute to the unidirectional movement of the substrates further facilitates the separation of distal and proximal binding pockets (Eicher et al., 2012; Zwama et al., 2018; Tam et al., 2020). As per the previous AcrB structural studies, the high molecular mass drugs, such as erythromycin and rifampicin, co-crystallize within the proximal binding pocket; however, the distal binding pockets are found to be responsible for holding both the low and high molecular mass drugs such as minocycline, doxorubicin, and neopentyl glycol derivative C7NG (Nakashima et al., 2011; Ababou and Koronakis, 2016; Sakurai et al., 2019). AcrB in *E. coli* and MexB homolog in *P. aeruginosa* and have similar structures and substrate-binding sites; therefore, both have similarities in a broad substrate specificity as well. Distal binding pocket (DBP) is a huge chamber surrounded by hydrophobic phenylalanine (Phe) residues and polar residues (Vargiu et al., 2018); further, the side chains present on these hydrophobic residues interact with the substrates through Pi–Pi stacking and van der Waals interaction, and on the contrary, polar residues interact *via* H-bonding. There are several pieces of evidence supporting the manifestation of broad substrate specificity of the pump as demonstrated by the different binding positions adopted by minocycline and doxorubicin. Moreover, according to previous structural studies, the role of a switch loop containing Gln 616–Gly 619 in regulating substrate's entrance into the distal binding pocket (Vargiu et al., 2018) and also in functioning as a barrier to separate the distal binding pocket from proximal pocket has been observed. Additionally, it has been suggested that physical contact between the substrate with the switch loop is essential for the translocation of the substrate before leaving the proximal binding pocket, and the movement instigated *via* the flexible loop facilitated the transportation of the substrates deep inside the distal binding site; however, without passing through the loop, the extrusion of the substrates *via* TolC is not possible (Nakashima et al., 2011; Eicher et al., 2012; Vargiu and Nikaido, 2012; Jamshidi et al., 2018). Immediately, after the binding of the substrate into the distal binding pocket occurs, it leads to a conformational change in the protein from state T to O and subsequently, the disintegration of tunnels 1 and 2 because of a transition of the coil to helix at N-terminal end of TM8 and extensive reorientation of carrier domains has been noticed (Zwama et al., 2017). Furthermore, for substrate transportation, a third tunnel that directs the substrates from AcrB on the way to TolC for extrusion is created in the state O (Eicher et al., 2012). Besides AcrB, the role of AcrA is also very crucial as repacking of AcrA helps to inhibit the leaking of the substrate into the periplasm *via* closing the cavities that exist inside the pump, and further, the changes arising in AcrA which result into the opening of the closed base of TolC are obviously initiated *via* the ligand binding phase due to the conformational changes occurring in AcrB. In addition, if the assemblage is exposed to the periplasm, it will restrict the opening of the TolC channel and the absence of any substrate inside a pump or extrusion of a substrate facilitates the conformational changes in AcrB from the drug transportation state to the resting state L (Wang Y. et al., 2017; Wang Z. et al., 2017). According to the aforementioned discussion regarding the homotrimeric symmetrical structure of AcrB which crystallizes immediately and forms substrate or inhibitor bound protein co-complexes because of their flexibility and multiple binding site mechanisms of AcrB, the movement for transportation of the substrates driven *via* multiple structural changes has been observed (Sakurai et al., 2019). A well-organized protein crystal required for the investigation of x-ray diffraction patterns can be difficult to grow when either of these processes is involved. However, currently, a number of compound-bounded AcrB structural studies have been carried out, which facilitated our understanding of the molecular foundation of inhibition (Murakami et al., 2006; Sennhauser et al., 2009; Nakashima et al., 2011, 2013; Eicher et al., 2012; Sjuts et al., 2016; Wang Y. et al., 2017; Wang Z. et al., 2017). Distal binding pockets (DBP) contain two characteristics crucial to inhibitor binding: (i) channels that translocate hydrophilic substrates and bind multiple drugs and (ii) a hydrophobic catcher that is located proximate to the distal binding pocket that divides this channel, which gives more attention to AcrB targeted small molecule inhibitors designing.

## Efflux pumps influencing biofilm foundation and quorum sensing

Besides the contribution of efflux pumps in the development of antibiotic resistance, some other mechanisms such as biofilm formation, which is clearly a community of microbes affixed to a surface, also contribute to the progress of bacterial resistance and tolerance; additionally, it has been suggested that the efflux pump system has the capability to influence the function of biofilm either directly or indirectly (Hall and Mah, 2017; Alav et al., 2018). In *A. baumannii*, biofilm formation is inhibited due to tigecycline exposure in sub-inhibitory concentration *via* the downregulation of the adeG gene encoding for efflux pumps (Chen et al., 2017). Quorum sensing (QS) is a network that is generated within cells to encourage mutual communication at the cellular level, and the utilization of signal transduction for participation in activities occurring inside bacteria has already been observed. The association between biofilm and QS improves bacterial viability in response to different variations in environmental signals. As an example, the ejection of acylated homoserine lactone (AHL) *via* the efflux pump system MexAB-OprM in *P. aeruginosa* is maintained by the QS contribution and overexpression of the efflux pump, which ultimately resulted in the release of QS signals (Piddock, 2006b). According to the available literature, it has been assumed that the bacterial QS mechanism is restricted if inhibitors hinder the efflux pump activity (Gupta et al., 2017). Similarly, in *S. aureus*, the inhibition of the NorA efflux pump activity due to the presence of an appropriate concentration of efflux pump inhibitor has also been described in a previous study (Sabatini et al., 2017). Additionally, the role of efflux pump systems in facilitating the pathogens in adapting to the harsh environment present within a mature biofilm, which involves response against envelope stress in maintaining the membrane rigidity and in conferring resistance toward high osmotic stress (Robin et al., 2022), such as the essential role played by the MacAB-TolC efflux pump in biofilm formation (Robin et al., 2022), has been reported. Pmt and AbaF are the two well-known MFS transporter proteins found in *A. baumannii*, which play an important role in the formation of biofilms such as Pmt in extracellular DNA extrusion and active participation of AbaF in emancipating biofilm materials (Pasqua et al., 2021). There is evidence indicating variations in bacterial membrane influencing the functioning of biofilm formation mediated *via* different MDR efflux pump systems belonging to the RND family (Yoon et al., 2016), as shown in *A. baumannii*, where the inhibition of adeAB gene expression or deletion resulting in reduced expression or hindrance of biofilm and QS systems have been described earlier (Richmond et al., 2016; Dou et al., 2017). A previous study proposed the incidence of a positive association between mRNA levels of efflux pump genes and biofilm formation, which can be influenced by the MIC levels of antibiotics such as polymyxin B or colistin (Sato et al., 2018). For example, MexGHI, an efflux pump system recognized in *P. aerugiosa* responsible for phenazine transportation, plays a crucial role in biofilm morphogenesis (Sakhtah et al., 2016). Similarly, the QS system also has a similar correlation with the efflux pump system (Liang et al., 2016). Additionally, a study evidenced the mutation in mexI, an efflux pump gene of *P. aeruginosa*, mediating the loss and reduced expression of quorum sensing molecules or virulence factors (Wolloscheck et al., 2018). The role of ATP-binding cassette (ABC) type efflux pump systems in conferring resistance toward antifungal agents that are used against fungi, mostly *Candida* species, is well known and their involvement in QS molecules secretion and influential capability toward biofilm formation is found to be similar to that of RND family members (Cannon and Holmes, 2015).

## Role of efflux pump inhibitors toward antimicrobial resistance

Efflux pumps largely contribute toward multidrug resistance, both intrinsic and extrinsic in bacteria; therefore, the active participation of efflux pump inhibitors (EPIs) have a potential impact in preventing antimicrobial resistance globally. A significant challenge in the discovery of EPI has been its highly hydrophobic nature and the substrate-binding poly-specific site for the target. However, the primary studies on the x-ray crystal structures of different compounds such as pyranopyridine (Sjuts et al., 2016) and pyridopyrimidine (Nakashima et al., 2013) (Table 1) revealed that the EPIs bound to different efflux pumps of RND family of *P. aeruginosa* and *E. coli* have helped to facilitate the rapid advancement in our understanding of different mechanisms involved and the binding interactions of these EPIs. Moreover, a strong binding interface between the Phe-abundant hydrophobic trap and the inhibitor D13-9001 participates in inhibiting the conformational change from state L to T and further hinders the functional rotation mechanism. Further, it has been suggested that the entry of the substrates into the binding cleft situated between the domains PC1 and PC2 is blocked by the hydrophilic moiety existing on the inhibitor D13-9001. Phe 178 is an imperative amino acid that exists in the hydrophobic trap, which plays a vital role in the binding of inhibitors with the aromatic substrates *via* utilizing π-π stacking (Nakashima et al., 2013; Sjuts et al., 2016), and it has been reported that, by applying a structure-guided design for an effective inhibitors development, the hydrophobic trap discovery has a major role (Aron and Opperman, 2018). As these efflux pumps are considered excellent targets for antimicrobial combination treatment, facilitating the usage of synthetic (Table 1) or plant-based (Table 2) efflux pump inhibitors (EPIs) to support antimicrobial therapeutic options toward bacterial infections (Opperman and Nguyen, 2015; Alibert et al., 2017). There are several effective approaches to restrict or avoid the activity of an active efflux pump system found in bacteria such as decreasing the antibiotic binding affinity toward transporters *via* chemical structural modification of the drug, escalating the OM permeability to increase the drug concentration within cell, knocking out or impeding the efflux pump associated genes, and disrupting the supply of ATP or *via* designing compounds which have the capability to compete with the antimicrobial agents for active sites for their action to obstruct the efflux pump activity (Jamshidi et al., 2016). Additionally, approaches such as computational analysis or artificial abstraction from plants were utilized for the detection of numerous inhibitors active against both Gram-positive and Gram-negative bacteria. Researchers working on screening different natural EP inhibitor compounds *via* utilizing high-throughput methods have observed a natural compound referred to as *Terminalia chebula*, which is isolated from an Indian medicinal plant, having the ability to abolish the binding of ligands with Na+, which resulted in a change in the conformation of the transporter protein NorM found in *N. gonorrhea* to closed state (Kesherwani et al., 2017). In *E. coli, P. aeruginosa*, and *A. baumannii*, one of the most widely studied efflux pump inhibitors of AcrB, phenylalanyl-arginine-β-naphthylamide (PAβN), is recognized for its ability to bind with the hydrophobic pocket located within AcrB, further inhibiting the drug extrusion activity (Lomovskaya et al., 2001; Ribera et al., 2002; Kinana et al., 2016). However, few other EPIs (MBX inhibitors) having the ability to bind strongly to AcrB have also been reported (Sjuts et al., 2016). Additionally, this inhibitor can be combined with a secondary metabolite, i.e., carolacton produced by mycobacteria, which can be utilized as a potential therapeutic option against pathogens over-expressing the AcrAB-TolC efflux pump system (Donner et al., 2017). Another example of EPIs is tannic acid, which inhibits the multidrug efflux pump systems, such as Tet and Msr (Tintino et al., 2017), and is well studied in *S. aureus*. Tannic acid has also been reported to reduce the MIC levels of different antibiotics such as erythromycin and tetracycline significantly (Tintino et al., 2017). However, in clinical settings, the usage of an antibiotic along with an efflux pump inhibitor (EPI) as combination therapy is a potential challenge that depends upon the internal permeability characteristics of the bacterial outer membrane. Further, in a study done by Yang et al. (2017) the use of combination therapy against multidrug-resistant *P. aeruginosa* comprising tobramycin and EPI (NMP, paroxetine, or DBP) encourages the binding of EPI with tetracycline; additionally, the response of the combination of tobramycin plus EPI along with rifampicin, fluoroquinolone, and fosfomycin was also analyzed, which revealed that they exert a robust effect cooperatively upon the bacteria, which resulted into reduction in the MIC80 level against these antibiotics (Yang et al., 2018). Another example of combination therapy containing levofloxacin and EPI (trimethoprim and sertraline) used against *P. aeruginosa* harboring the MexAB-OprM efflux pump system showed a progressive advantage over monotherapy (using levofloxacin alone) (Adamson et al., 2015). Similar research conducted by Prasch and Bucar (2015) has also validated that a reduction in the antibiotic dose was observed when the combination therapy comprising EPI inhibitor and antibiotics was co-administered. Besides the aforementioned EPIs, several plant-based EPIs, have also been investigated, and above 20 different EPIs derived from plants have been reported based on their extraction mechanisms (Garvey et al., 2011b; Shiu et al., 2013). Additionally, vegetable-derived compounds, such as artesunate, berberine (Table 2), and curcumin, have also been reported to have the ability to inhibit EP activity in *E. coli* and *P. aeruginosa*, besides their anti-inflammatory and antibacterial activities (Li et al., 2011; Negi et al., 2014; Laudadio et al., 2019). Different food items such as vegetables, including *Momoedica balsamina*, spices, such as cumin and pepper, and oils extracted from aromatic plants are known for being an exceptional source of EPIs (Karumathil et al., 2018; Li et al., 2019; Tariq et al., 2019; Solnier et al., 2020; Muniz et al., 2021); further, the application of flavonoids such as flavonolignans in combating MDR *via* inhibiting efflux pump systems have been described previously (Chambers et al., 2019).

**Table 1:** Examples of synthetic efflux pump inhibitors (EPIs) having inhibitory action against Gram-negative bacteria.

**S. No**	**Efflux pump inhibitor (EPI) types**	**Efflux pump substrate (s)**	**Effective against bacteria**	**References**
1	Peptidomimetics	Chloramphenicol, fluoroquinolones, macrolides, carbapenem, tetracyclines	*E. coli, P. aeruginosa, K. pneumoniae, S. enterica, Campylobacter* spp., *E. aerogenes, A. baumanii*	Renau et al., 1999; Malléa et al., 2002; Mamelli et al., 2003; Lomovskaya and Bostian, 2006; Cortez-Cordova and Kumar, 2011; Vera-Leiva et al., 2018
2	Quinoline derivatives	Chloramphenicol, tetracycline, norfloxacin	*E. aerogenes, K. pneumoniae*	Chevalier et al., 2001; Gallo et al., 2003; Malléa et al., 2003; Ghisalberti et al., 2006; Mahamoud et al., 2006
3	Trimethoprim	Chloramphenicol, tetracycline, ciprofloxacin, erythromycin	*Enterobacteriaceae, P. aeruginosa*	Piddock et al., 2010
4	Aminoglycoside analogs	Tetracycline, gentamicin	*H. influenza*	Van Bambeke and Lee, 2006
5	Microbial EPIs	Levofloxacin	*P. aeruginosa*	Lee et al., 2001
6	Arylpiperazines	Chloramphenicol, tetracyclines, fluoroquinolones, macrolides, linezolid	*A. baumanii, P. aeruginosa, C. jejuni Enterobacteriaceae*	Bohnert and Kern, 2005; Kern et al., 2006; Pannek et al., 2006; Schumacher et al., 2006; Kurinčič et al., 2012; Sonnet et al., 2012
7	Indole derivatives	Chloramphenicol, erythromycin, ciprofloxacin, tetracycline	*E. coli*	Zeng et al., 2010
8	sRNA and antisense oligonucleotides	Ciprofloxacin, erythromycin	*E. coli, C. jejuni*	Van Bambeke and Lee, 2006; Mu et al., 2013
9	Arylpiperidines	Linezolid	*E. coli*	Thorarensen et al., 2001
10	Substituted polyamines	Unknown	*H. influenza*	Mahmood et al., 2016
11	Hydantoins	Chloramphenicol, nalidixic acid, sparfloxacin, doxycycline, erythromycin	*E. coli, E. aerogenes*	Handzlik et al., 2011; Otreebska-Machaj et al., 2016
12	Epinephrine	Chloramphenicol, tetracycline, ciprofloxacin, erythromycin	Enterobacteriaceae, *P. aeruginosa*	Piddock et al., 2010
13	Antibiotic analogs, tetracycline analogs	Tetracyclines	*E. coli*	Nelson et al., 1993, 1994
14	Quinazoline derivatives	Chloramphenicol, nalidixic acid, sparfloxacin	*E. aerogenes, P. aeruginosa*	Chevalier et al., 2010
15	Pyranopyridines	Fluoroquinolones, piperacillin	Enterobacteriaceae	Opperman et al., 2014; Vargiu et al., 2014; Aron and Opperman, 2016; Sjuts et al., 2016
16	Phenothiazines	Chloramphenicol, tetracyclines, nalidixic acid, levofloxacin, triclosan, erythromycin, aminoglycosides	*E. coli, S. enterica, B. pseudomallei*	Chan et al., 2007; Bailey et al., 2008; Martins et al., 2008
17	Serum compounds	Minocycline, ciprofloxacin	*A. baumanii, P. aeruginosa*	Blanchard et al., 2014
18	Antibiotics globomycin	Chloramphenicol, norfloxacin	*E. aerogenes*	Malléa et al., 2002
19	Fluoroquinolone analogs	Fluoroquinolones, macrolides	*E. coli, P. aeruginosa*	Van Bambeke and Lee, 2006
20	Pyridopyrimidines	Fluoroquinolones, B-lactams	*P. aeruginosa*	Yoshida et al., 2006, 2007
21	Naphthamides	Chloramphenicol, tetraphenylphosphonium, erythromycin	*E. coli*	Wang Y. et al., 2017; Wang Z. et al., 2017; Wang et al., 2018

**Table 2:** List of plant-derived efflux pump inhibitors (EPIs) having inhibitory action toward Gram-negative bacteria.

**Plant**	**Compound extracted**	**Effective against**	**References**
*Ipomoea muricata*	Lysergol	*E. coli*	Maurya et al., 2013
*Glycine max*	Daidzein	*E. coli*	Dwivedi et al., 2015
*Baccharoides adoensis, Callistemon citrinus*	Ethanolic extract	*Pseudomonas aeruginosa*	Chitemerere and Mukanganyama, 2014
*Larrea tridentata*	Nordihydroguaretic acid	*E. coli*	Ohene-Agyei et al., 2014
*Lithospermum erythrorhizon*	Shikonin		
Plumbagin	*Plumbago indica*		
*Digitalis lanata*	Lanatoside C	*E. coli, Pseudomonas aeruginosa*	Aparna et al., 2014
Acer saccharum	Phenolic-rich maple syrup extracts (PRMSE)	*E. coli, Proteus mirabilis, Pseudomonas aeruginosa*	Maisuria et al., 2015
*Ammannia* spp.	4-Hydroxy-alpha-tetralone + semisynthetic derivatives	*E. coli*	Dwivedi et al., 2014
Eucalyptus tereticornis	Ursolic acid	*E. coli*	Dwivedi et al., 2015
*Berveris bulgaris*	Berberine, palmatine	*Pseudomonas aeruginosa*	Aghayan et al., 2017
Holarrhena antidysenterica	Conessine		Siriyong et al., 2017

However, incidences of another resistance mechanism associated with membrane-embedded MDR efflux pumps which can extrude a wide range of noxious compounds such as antibiotics, dyes, and biocides have been reported (Mitchell et al., 2019; Jesin et al., 2020), which can be a promising novel approach for tackling the increasing risk of antibiotic-resistant bacteria. Based on previous studies, all the bacterial species possess MDR efflux pumps, conferring intrinsic resistance against antibiotics (Bay and Turner, 2009), and six different superfamilies of efflux pumps have already been reported, but mostly, the small multidrug resistance efflux pump family was studied extensively (Sun et al., 2014). In hospitals, the commonly used disinfectants, the quaternary ammonium compounds (QACs), toxic compounds such as ethidium bromide, and many other compounds such as biocides, cetyltrimethylammonium bromide (CTAB), and benzalkonium chloride (BZK) are utilized as a substrate for acquiring energy by the SMRs family members (Bay et al., 2008). A study by Kadry et al. (2017) revealed the existence of a strong correlation between the gene encoding SMR protein with the enhanced level of resistance toward compounds such as benzalkonium chloride in clinical isolates of *P. aeruginosa*.

## Conclusions

In this review study, we have discussed the important role played by efflux pump family members in developing resistance against single or multiple antibiotics as well as against various compounds other than the antimicrobial agents including heavy metals, preservatives, and toxins. Apart from different classes of antibiotics, efflux pumps also provide resistance against disinfectants and virulence factors and even develop cross-resistance against other different noxious compounds, which further leads to the development of a multidrug-resistant phenotype. The working mechanism along with the regulation of an efflux pump system is an intricate process that actively participates in different biological processes in bacteria such as biofilm formation, quorum sensing, and adhesion of bacteria to host, and their progressive multiplication have been highlighted in this review. This review further highlights the importance of the discovery of novel EPIs, which should be plant-based, safe, and non-toxic, and many efflux pump inhibitors that are under clinical trial as well. Efflux pump inhibitors derived from plants are of great importance and are studied widely presently. However, the variations in function and regulatory mechanisms of the efflux pump systems along with the progress and utilization of a variety of efflux pump inhibitors still need further exploration. According to the progressive research work, it has been suggested that TetR family members can be studied further for the discovery of antibiotic residues associated with multidrug resistance. In addition to the role of the aforementioned active regulatory proteins, some other proteins with an ability to identify single or multiple substrates and specific substrate-binding domains that can be utilized to identify antibiotic residues involved in substrate binding have been reported earlier. Therefore, other than the TetR regulatory protein, the detection of residues of antibiotics and transcriptional regulatory protein requires the utilization of efflux pump concomitant genes. Additionally, the proteins with a high substrate binding specificity toward diverse antimicrobials with more appropriate features are very demanding. Moreover, this review discussed the association of the expressional levels of efflux pump systems with various other defense mechanisms such as biofilm formation and quorum sensing, which provides novel insights for expanding the extent of fundamental research. In this article, we provided an overview of efflux genes and significant transporters indicating an area which requires persistent exploration to provide supervision for clinical practices.

## Author contributions

SC was involved in the literature review, writing of the original draft, figure illustrations, conceptualization, preparing the draft of the manuscript, reviewing, editing, revising the manuscript, has contributed to the article, and approved the submitted version.
